# Severity of Depression, Anxious Distress and the Risk of Cardiovascular Disease in a Swedish Population-Based Cohort

**DOI:** 10.1371/journal.pone.0140742

**Published:** 2015-10-15

**Authors:** Aysha Almas, Yvonne Forsell, Romaina Iqbal, Imre Janszky, Jette Moller

**Affiliations:** 1 Karolinska Institutet, Department of Public Health Sciences, Stockholm, Sweden; 2 Department of Medicine, Aga Khan University, Karachi, Pakistan; 3 Department of Community Health Sciences and Medicine, Aga Khan University, Karachi, Pakistan; 4 Departments of Public Health and General Practice, Faculty of Medicine, Norwegian University of Science and Technology, Trondheim, Norway; Chiba University Center for Forensic Mental Health, JAPAN

## Abstract

**Background:**

Depression is known to be associated with cardiovascular diseases (CVD). This population-based cohort study aimed to determine the association between depression of varying severity and risk for CVD and to study the effect of concomitant anxious distress on this association.

**Methods:**

We utilized data from a longitudinal cohort study of mental health, work and relations among adults (20–64 years), with a total of 10,443 individuals. Depression and anxious distress were assessed using psychiatric rating scales and defined according to DSM-5. Outcomes were register-based and self-reported cardiovascular diseases.

**Findings:**

Overall increased odds ratios of 1.5 to 2.6 were seen for the different severity levels of depression, with the highest adjusted OR for moderate depression (OR 2.1 (95% CI 1.3, 3.5). Similar odds ratios were seen for sub-groups of CVD: ischemic/hypertensive heart disease and stroke, 2.4 (95% CI 1.4, 3.9) and OR 2.1 (95%CI 1.2, 3.8) respectively. Depression with anxious distress as a specifier of severity showed OR of 2.1 (95% CI 1.5, 2.9) for CVD.

**Conclusion:**

This study found that severity level of depression seems to be of significance for increased risk of CVD among depressed persons, although not in a dose-response manner which might be obscured due to treatment of depression. Further, we found a higher risk of CVD among depressed individuals with symptoms of anxious distress.

## Introduction

Depression is a large and increasing public health problem, and the majority of cases are not detected in health care.[1] It is associated with increased risk of overall mortality and morbidity such as cardiovascular-related outcomes, Alzheimer’s disease, obesity and cancer.[2]

Studies have been conducted to establish the temporal relationship between depression and cardiovascular-related outcomes.[3–9] In a meta-analysis including 146,538 participants from 54 observational studies, Nicholson et al reported that it was difficult to establish whether depression is a causal factor for coronary heart disease.[10] Studies need to be designed with appropriate methodology to clarify the independent impact of interventions and comorbid disease on outcomes.[11] Depression has also been associated with stroke.[12,13] However there is lack of data from studies where depression has been assessed by means of questionnaires validated by interviews, thus ensuring that the symptoms are not purely part of a somatic disorder. The dose-response relationship between depression and future risk of CVD has not been well studied. So far there is are two studies that report incremental increase in the risk of future CVD with increasing severity of depression. However both studies are based on patients from hospital or clinic setting.[14,15]

More than half of the persons affected by depression also have symptoms of anxious distress, which is regarded as an indicator of severity of depression as it has been associated with increased functional impairment, greater overall illness chronicity, and increased risk of suicide.[16] Due to this clinical importance, anxious distress has been added as a specifier for depressive disorders in the Diagnostic and Statistical Manual of Mental Disorders-5 (DSM-5) giving the clinician the opportunity to rate not just the presence of such symptoms but also their degree of severity. Anxious distress as a specifier of depression has not been studied in relation to the future risk of CVD. Although there are studies on the association of anxiety as a separate disease entity with CVD [17,18], less is known about whether the combination of depression and anxious distress symptoms imposes a different risk of CVD than depression alone.

This population-based cohort study aims to determine the association between depression and risk for CVD, and to study the effect of severity of depression and concomitant symptoms of anxious distress on this association.

## Methods

### Population and sample

#### Study design

We utilized data from the PART study (In Swedish short for: Psykisk hälsa, Arbete och RelaTioner) which is a longitudinal cohort study of mental health, work and relations among adults (20–64 years) residing in Stockholm County, Sweden.[19] The PART study has had three measurement points: wave 1 (W1) in 1998–2000, wave 2 (W2) in 2001–2003 and wave 3 (W3) in 2010. During each wave, participants answered a postal questionnaire dealing with the risk and protective factors of mental health and psychiatric rating scales. In the present study, all participants from W1 were followed up in W3 for occurrence of cardiovascular diseases through self-reported outcomes and data in the National Patient Register (NPR) during 2008–2011.[20]

The Ethical Review Board at Karolinska Institutet, Stockholm, approved the study (case number: 2010/1185-31/596:260, 01–218, 04-528/3 and 09–880). After a complete description of the study to the subjects, written informed consent was obtained.

The PART study aimed to include 19,744 persons but only 19,457 could be reached, and 10,443 individuals responded to the questionnaire at W1 (participation rate 53%). Non-response analyses have been performed using available administrative registers, and participation was seen to be related to female gender, higher age, higher income and education, being born in the Nordic countries and having no previous psychiatric diagnosis in inpatient registers.[21]

All participants received a second and third, almost identical questionnaire, three and ten years after they had answered the first one (participation rate 83%, n = 8,622 in W2 and 61%, n = 5,228 in W3). Attrition in the second wave was associated with similar factors to those in the first wave. Additionally, participants with previous psychiatric hospital discharge diagnosis at W1 had a higher probability of non-participation at W2 (OR 1.6 (95% CI 1.1, 2.4).[22] A description of the study sample is given in “Table 1”.

**Table 1:** Characteristics of the participants overall, and stratified by depression status.

	Overall	Depressed^1^	Not depressed	P value ^2^
	N = 10341	N = 1488 (14.42)	N = 8832 (85.58)	
	N (%)	N (%)	N (%)	
**Mean age (SD) in years**	41.3 (12.4)	39.56 (11.9)	41.67 (12.5)	<0.001
**Male gender**	4620 (44.0)	463 (31.1)	4146 (46.9)	<0.001
**Socio-economic position**				
High and intermediate level salary	4273 (41.2)	579 (52.4)	3679 (54.6)	
Assistant –non manual workers	1359 (13.1)	197 (17.8)	1152 (17.1)	
Skilled workers	609 (5.9)	79 (7.2)	529 (7.9)	
Unskilled and semiskilled workers	991 (9.6)	146 (13.2)	832 (12.4)	
Self-employed (other than professional)	649 (6.3)	103 (9.3)	543 (8.1)	0.38
**IHD**	193 (1.9)	47 (3.2)	143 (1.6)	<0.001
**Stroke**	86 (0.8)	23 (1.5)	63 (0.7)	0.003
**Hypertension**	716 (6.9)	126 (8.5)	590 (6.7)	0.01
**Diabetes mellitus**	221 (2.1)	38 (2.6)	181 (2.0)	0.20
**Smoking**				
Regular	1289 (12.4)	306 (24.5)	983 (13.6)	
Occasional smoker	889 (8.6)	148 (11.9)	741 (10.3)	
Ex-smoker	2502 (24.1)	331 (26.5)	2171 (30.0)	
Never smoker	3796 (36.6)	462 (37.0)	3333 (46.1)	<0.001
**Physical activity**	4574 (44.1)	550 (44.1)	4024 (55.4)	<0.001
**Mean BMI (SD) in kg/m2**	24.97 (3.9)	25.13 (4.4)	24.93 (3.7)	0.1
**Hazardous alcohol use**	2605 (25.2)	544 (36.6)	2060 (23.3)	<0.001
**Sought treatment for psychiatric disorders**	2365 (22.8)	821 (55.2)	1542 (17.5)	<0.001
**Severity of depression (MDI)** ^**1**^				
Mild	-	534 (5.1)	-	
Moderate	-	367 (3.5)	-	
Severe	-	587 (5.6)	-	
**Anxious distress symptoms**				
Mild	1729 (16.7)	539 (36.3)	1189 (13.5)	
Moderate/severe	793 (7.7)	628 (42.3)	165 (1.9)	<0.001
**Cardiovascular diseases at follow-up**	514 (5)	96 (6.5)	415 (4.7)	0.002
**Ischemic/hypertensive heart at follow-up**	364 (3.5)	64 (4.3)	297 (3.4)	0.04
**Stroke at follow-up**	228 (2.2)	49 (3.3)	179 (2)	0.001

^1^ Depression assessed by Major Depression inventory,

^2^Pearson chi square test and independent sample t test were used as appropriate.

### Depression

Depression was assessed using the Major Depression Inventory (MDI) based on responses given in W1 or W2. For those who were depressed in both W1 and W2, the wave in which they scored highest MDI was used to assess level of severity. The MDI has shown high validity in both clinical and non-clinical samples, including the PART study [23–25] and can also be used as a diagnostic instrument with the algorithms for DSM-IV. The MDI scale comprises 10 questions on symptoms present nearly every day during the previous two weeks. Each question has five response alternatives scored from 1–5 according to the presence of the symptom; all the time (5), most of the time (4), slightly more than half of the time (3), slightly less than half of the time (2), some of the time (1) and never (0). The sum score of all 10 questions ranges from 0–50.[26] In order to validate the MDI scale a subsample was interviewed by psychiatrists using Schedules for Clinical Assessment in Neuropsychiatry. The study concluded that the MDI score had a relatively high sensitivity (67%) for diagnosis of major depression.[23]

#### Severity of depression

Severity of depression was based on the MDI score and categorized as follows: not depressed (MDI score < 20), mild depression (20–24), moderate (25–29) and severe depression (MDI ≥ 30).[27]

*Anxious distress*: Anxious distress was defined according to DSM-V criteria and used as a specifier for depression. This specifier is recommended to be used along with depression to grade severity of depression.[28] The PART study was extensive and included multiple scales to assess mental health. Anxious distress was based on specific items Appendix A in S1 Table) corresponding to the DSM-V from these scales. We combined the moderate-severe category with severe as the severe category includes assessing motor agitation which requires a clinical observation.[29] Anxious distress was defined as the presence of at least two of the following symptoms during the previous two weeks: feeling keyed up or tense, feeling unusually restless, difficulty in concentrating because of worry, fear that something awful might happen and fear of losing self-control. Each symptom was assessed using a 5- or 6-point Likert scale, and, for simplicity, the response alternatives were merged as yes or no, and rated according to DSM-5 number of symptoms (no anxious distress = none or one symptom; mild anxious distress = two symptoms; moderate anxious distress = three symptoms; and moderate to severe anxious distress = four to five symptoms)

### Cardiovascular diseases

Cardiovascular disease (CVD) was assessed by self-reported health status questions in W3.”Are you or have you previously been on treatment for angina pectoris or have you received this diagnosis from a physician? “Corresponding questions were asked for myocardial infarction, high blood pressure and stroke. In addition, hospital discharge diagnoses in the National Patient Register (NPR) 2008 to 2011 were examined. The following diagnoses were coded in the NPR according to ICD10 [30,31]: ischemic/hypertensive heart disease; hypertensive diseases (I11-13), ischemic heart diseases (I20-25), heart failure (I50), other peripheral vascular diseases, embolism and thrombosis (I73-74) and stroke (I60-67 and I69).

### Covariates

The following variables were selected as potential confounders: age, gender, socioeconomic position (SEP), history of ischemic heart disease, hypertension, stroke and diabetes at baseline (W1). The reason to consider these as confounders was that they are associated with the exposure depression and cardiovascular outcomes and do not to come in the causal pathway (when considered at baseline) between depression and CVD.[32–34] History of ischemic heart disease and stroke were measured at W1 and were considered as previous history of CVD and the final models were adjusted for them. We considered smoking, hazardous alcohol use, BMI and physical activity as mediators since they are established risk factors for both depression and CVD and are also likely to come in the causal pathway. [35–40] Age at W1 was categorized into 30–45 years, 46–60 years, and 61–70 years and >71 years. SEP in W1 was measured through occupational groups defined according to the Nordic Standard Occupational Classification (NSOC) of 1989.[41] SEP was classified into five groups: high/intermediate level salaried employees; assistant non-manual employees; skilled workers; unskilled workers; and self-employed (including farmers). Participants were considered as physically active if they reported exercise regularly at least three times a week. Smoking habits reported in W2 was classified as regular smoker, occasional smoker, previous smoker and never smoker.[42] Hazardous alcohol use in W1 and W2 was assessed by the Alcohol Use Disorders Identification Test (AUDIT) tool [43] and dichotomized following the Swedish cut-off points (≥8 points for men and ≥6 points for women).[44]. Treatment for psychiatric disorders was recorded through questions inquiring whether the participant had sought psychiatric consultation in either W1 or W2. For those variables for which data was available from both W1 and W2, a combined variable was created for both waves indicating presence in both waves or maximum value in either wave for a continuous variable (e.g. body mass index (BMI) and hazardous alcohol intake).

### Statistical Analyses

Descriptive analyses were reported as mean and standard deviation (SD) for continuous variables and frequency and percentage for categorical variables. Imputation of missing values for MDI and DSM-IV was performed when there were missing answers for one or two of the ten questions. If answers were missing for more than two questions, the response was left as missing. The missing answers were imputed with the mean value of the questions in the answered items. The number of missing answers for three or more questions was low, 0.2% (n = 23) in W1 and 1.4% (n = 121) in W2. A similar strategy was used for imputation of anxious distress although only for one missing answer. Missing values for other variables were treated as such in the analyses.

Independent sample t-tests and chi-square tests were used to compare the depressed and non-depressed. Logistic regression was used to calculate odds ratios (OR) and corresponding 95% confidence intervals (95%CI) for the association between depression and CVD, and adjusting for confounders. We used those without depression in the main analyses as a reference group and for the sub-analyses including anxious distress, we used those with neither depression nor anxious distress. The test of homogeneity for OR (Breslow-Day with Tarone's adjustment) was used to determine if the association of depression with CVD differed across different strata of anxious distress. SPSS version 19.11 and SAS 9.3 were used for the statistical analyses.

## Results

The prevalence of depression, according to MDI, was 14.4% (n = 1488): 5.2% (n = 534) had mild, 3.5% (n = 367) had moderate and 5.7% (n = 587) had severe depression. Applying the DSM-IV algorithm, the overall prevalence of depression was 7.6% (n = 790). Persons affected by depression were slightly younger and more often females than those not affected. Persons with severe depression reported seeking treatment for psychiatric disorders more often 16.9% (n = 400) than those with moderate 8% (n = 189) or mild 9.8% (n = 232) depression. During follow-up, the risk of CVD was 5% (n = 514), of which ischemic/hypertensive heart diseases was 3.5% (n = 364), stroke was 2.2% (n = 228) and 0.8% (n = 78) had both ischemic/hypertensive heart disease and stroke.

Depression was associated with risk for CVD (OR 1.9 (95% CI 1.4, 2.5). The association weakened somewhat after taking confounding factors into account (OR 1.7 (95% CI 1.2, 2.3) but remained in the final model after adjusting for mediators (OR 1.5 (95% CI 1.1, 2.1)). Similar associations were found in CVD subgroups: with adjusted OR 1.5 (95% CI 1.0, 2.1) for ischemic/hypertensive heart diseases and OR of 1.7 (95% CI 1.1, 2.6) for stroke “Table 2”. The analysis was also done using the DSM IV algorithm for depression and the results were similar to those from MDI.

**Table 2:** Odds ratio for cardiovascular disease by depression status and severity of depression and anxious distress (n = 10,341).

	Cardiovascular diseases			Ischemic/hypertensive heart disease			Stroke		
	N = 514			N = 364			N = 228		
	Model 1^1^	Model 2^2^	Model 3^3^	Model 1^1^	Model 2^2^	Model 3^3^	Model 1^1^	Model 2^2^	Model 3^3^
	OR (95%)	OR (95%)	OR (95%)	OR (95%)	OR (95%)	OR (95%)	OR (95%)	OR (95%)	OR (95%)
**Depression status (MDI)**									
No	1.0 (Ref)	1.0 (Ref)	1.0 (Ref)	1.0 (Ref)	1.0 (Ref)	1.0 (Ref)	1.0 (Ref)	1.0 (Ref)	1.0 (Ref)
Yes	1.9(1.4,2.5)	1.7(1.2,2.3)	1.5(1.1,2.1)	1.8(1.3,2.5)	1.6(1.1,2.3)	1.5(1.0,2.1)	2.0(1.4,3.0)	1.8(1.2,2.7)	1.7(1.1,2.6)
**Level of depression (MDI)**									
No depression	1.0 (Ref)	1.0 (Ref)	1.0 (Ref)	1.0 (Ref)	1.0 (Ref)	1.0 (Ref)	1.0 (Ref)	1.0 (Ref)	1.0 (Ref)
Mild depression	1.5(0.98,2.5)	1.4 (0.8,2.3)	1.3 (0.8,2.2)	1.9 (1.1,3.3)	1.8 (1.0,3.1)	1.7 (1.0,3.0)	1.7 (0.9,3.3)	1.6 (0.8,3.1)	1.5 (0.7,3.0)
Moderate depression	2.6 (1.6,4.2)	2.3(1.4,3.8)	2.1(1.3,3.5)	2.1(1.2,3.8)	1.8(1.0,3.4)	1.7(0.9,3.3)	2.7(1.4,5.1)	2.4(1.2,4.7)	2.2(1.1,4.3)
Severe depression	1.7 (1.1,2.7)	1.5(0.9,2.4)	1.3(0.9,2.2)	1.4(0.8,2.5)	1.2(0.7,2.3)	1.1(0.6,2.0)	1.9(1.0,3.5)	1.6(0.9,3.1)	1.5(0.8,2.9)
**Depression (MDI) with/without symptoms of anxious distress**									
No depression or anxious distress	1.0 (Ref)	1.0 (Ref)	1.0 (Ref)	1.0 (Ref)	1.0 (Ref)	1.0 (Ref)	1.0 (Ref)	1.0 (Ref)	1.0 (Ref)
Depression without anxious distress	1.6 (0.8,3.0)	1.6(0.8,3.1)	1.5(0.7,2.9)	1.4(0.6,3.2)	1.6(0.7,3.5)	1.5(0.6,3.4)	2.2(1.0,4.8)	2.2(1.0,5.0)	2.1(0.9,4.7)
Depression with anxious distress	2.1 (1.5,2.9)	1.8(1.2,2.5)	1.6(1.1,2.3)	2.0(1.4,3.0)	1.7(1.1,2.6)	1.6(1.0,2.4)	2.0(1.3,3.1)	1.7(1.1,2.7)	1.6(1.0,2.6)
**Depression (MDI) with/without levels of anxious distress**									
No depression or anxious distress	1.0 (Ref)	1.0 (Ref)	1.0 (Ref)	1.0 (Ref)	1.0 (Ref)	1.0 (Ref)	1.0 (Ref)	1.0 (Ref)	1.0 (Ref)
Depression without anxious distress	1.5 (0.8,3.0)	1.6(0.8,3.1)	1.5(0.7,2.9)	1.4(0.6,3.2)	1.6(0.7,3.5)	1.5(0.6,3.4)	2.2(1.0,4.8)	2.2(1.0,5.0)	2.1(0.9,4.7)
Depression with mild anxious distress	2.7 (1.8,4.1)	2.3(1.5,3.5)	2.1(1.4,3.3)	3.0(1.9,4.7)	2.5(1.5,4.1)	2.4(1.4,3.9)	2.7(1.5,4.8)	2.3(1.3,4.1)	2.1(1.2,3.8)
Depression with moderate/severe anxious distress	1.6 (1.0,2.5)	1.3(1.4,2.3)	1.2(0.7,2.0)	1.2(0.7,2.3)	1.1(0.6,2.0)	0.9(0.5,1.8)	1.5(0.7,2.8)	1.2(0.6,2.5)	1.1(0.6,2.3)

^1^Model 1 adjusted for age and gender,

^2^ Model 2 adjusted for age, gender, SEP, history of IHD, stroke, hypertension and diabetes,

^3^Model 3 adjusted for age and gender, SEP, history of IHD, stroke, hypertension and, diabetes smoking, physical activity, BMI and hazardous alcohol consumption.

Overall increased odds ratios of 1.5 to 2.6 were seen for the different severity levels of depression, with the highest OR for moderate depression. The association seemed to be partly explained by confounders in model 2 and mediators in the final model for mild and severe depression whereas the OR for moderate depression remained with CVD (OR 2.1 (95% CI 1.3, 3.5).

Table 2 further shows the association between depression and symptoms of anxious distress and CVD. When we analyzed the effect of the combination of depression with anxious distress, increased OR of 2.1(95% CI 1.5, 2.9) was seen, which remained in the final model. When analyzing the effect of the combination of depression and level of anxious distress we found that depression in combination with mild anxious distress was associated with CVD (adjusted OR 2.1 (95% CI 1.4, 3.3). This was also confirmed by running a homogeneity test (p value = 0.17). Increased odds ratios were also seen for depression in combination with moderate/severe anxious distress, although this was not statistically significant. Similar associations for risk of CVD were also seen in the sub-groups of CVD, where adjusted OR for ischemic/hypertensive heart diseases was 2.4 (95% CI 1.4, 3.9) and for stroke was OR of 2.3 (95% CI 1.3, 4.1).

## Discussion

In this large cohort of 10,341 adults, we found an association between depression and the future risk of CVD, similar across sub-groups of CVD, but less strong when taking socio-demographic and lifestyle factors into account. We found that level of severity of depression seems to matter for increased risk of CVD, although not in a dose-response manner which might be obscured due to treatment of depression. Overall, moderately depressed persons showed higher estimates for CVD compared to mildly and severely depressed. Further, we found a higher risk of CVD among depressed who also suffered from anxious distress symptoms.

The association between depression and incident CVD has been reported previously, and our results are in line with previous findings although differences exist in population age and the instruments used for assessing depression.[45] A meta-analysis including 28 studies and comprising approximately 80,000 subjects concluded that depression is a risk factor for incident CVD.[46] Two meta-analyses on approximately 500,000 participants and 45 studies, published specifically on the association of depression as a risk factor for stroke, concluded that depression is associated with increased risk of incident stroke.[47,48] There have also been reports using Swedish populations previously. Rahman et al, for example, in an elderly Swedish population (selected and followed using the national inpatient register) reported a hazard ratio of 1.76 (95%CI 1.14, 2.71) for the association of depression with ischemic stroke but no association with coronary heart disease.[49] In contrast, we found an association of depression with hypertensive/ischemic heart disease and stroke. The reason for this might be that the population in our study was relatively younger and we took previous CVD related disorders into account. It is likely that, for example, hypertension was treated only in outpatient care and thus the information was not available in the registers. In a 37-year follow-up study of Swedish young men, an association was found between anxiety disorders and CVD but not with depression. However, as there was no intermediate assessment of participants in the above study during the long follow-up period, this might have diluted the exposure status of depression. Additionally, late onset depression was missed resulting in misclassification among participants with no depression[17].

Severity of depression and its association with future risk of CVD has been reported previously in two studies. In the Nova Scotia Health Survey–1995 (n = 1302) depression was assessed as a continuous score by the center for epidemiological studies–depression scale and found an independent, gradient effect between depression and incident coronary heart disease.[14] Further, Seldenrijk et al reported from the Netherlands Study of Depression and Anxiety (n = 2510), based on individuals visiting primary and mental health clinics, a dose-response association between severity of depression (measured by composite international diagnostic interview) and CVD; HR (95%CI) of 1.4 (0.7,2.5) for mild depression, 2.5 (1.2,4.5) for moderate depression and 3.04 (1.3,5.7) for severe depression.[15] In our study we did not find any clear dose response relation between depression and CVD. We think this potentially could be due to this study being population based with 10 year follow up, while the former two studies are based on hospital, primary and mental health clinics. Also in the former study the proportion of participants who were exposed to depression or anxiety was 78% while the corresponding number in our study was 14%. Also we hypothesize that people with severe depression seek treatment for psychiatric disorders more commonly than those with milder forms, which also was the case in the present study. It likely that persons who are treated for depression are also treated for somatic symptoms and advised to alter their life style thus reducing the risk factors for CVD.[50] We might have over-adjusted our results as smoking, physical activity and BMI might act as mediators rather than confounders, being risk factors for both depression and CVD.

Depression manifests clinically through metabolic, immune-inflammatory, autonomic and hypothalamic pituitary axis dysregulation all of which affect the incidence of CVD.[51] It is often accompanied by cognitive impairment, silent brain infarcts and hemorrhages which are linked to stroke.[52] Additionally brain-derived neurotrophic factor (BDNF) and sigma-1 receptor play a cardio- protective role in the pathophysiology of CVD and depression in animal models and could serve as targets for therapy.[53] We also know that some anti-depressants like the tricyclic antidepressants also increase the risk of cardiac arrhythmia.[54] In the present study, data on treatment did not take into account the compliance and type of treatment offered to the participants and thus might not be a true reflection of treatment status. On the other hand, people with mild or moderate depression who did not seek treatment might have increased risk of CVD.

We found that when anxious distress symptoms was present in depressed persons, it increased the future risk of CVD, although not in a dose-response manner and not as clearly for stroke. One potential explanation to the finding that the risk of future CVD was higher in patients who were depressed with mild anxious distress compared to those with moderate to severe anxious distress could be that the former tend to seek treatment more often.[50] This might lead to fewer of the depressed individuals with mild anxious distress progress to more morbidity. Studies on anxious distress as a severity indicator of depression have not been reported to date, however there are many studies on anxiety as a separate disorder and its relation to CVD. Some cross-sectional studies have reported associations between anxiety and CVD. [55–57]. In a longitudinal study by Gustad et al on 62,567 Norwegian adults, symptoms of anxiety were associated with increased risk of incident heart failure [18] which is in agreement with our study. However in the former study anxiety was assessed as a separate disease entity while we assessed anxious distress in depressed persons as an indicator of severity. In another prospective study of anxiety and incident stroke, on 6,019 participants from the First National Health and Nutrition Examination Survey (NHANES I), higher anxiety symptom levels were associated with an increased risk of incident stroke, independent of other risk factors, including depression.[58] That study assessed symptoms of anxiety using a generalized wellbeing schedule which is actually a validated scale for assessment of general well-being, while we have used validated scales for anxiety distress following DSM-5 criteria.

The strength of this study lies in its longitudinal design, the use of a population-based sample and the use of the MDI, which is a validated scale for measuring depression. It is one of very few studies on the level of severity of depression with concomitant anxious distress and future risk of CVD. Our study also has some limitations that have to be acknowledged. The response rates in W1 were 53%, which is quite low [21] and the participation rate at the follow-up phase (wave 3) 61% (n = 5,228). The follow-up rate in wave 3 was rather low leading to potential underestimation of CVD outcomes. However, to overcome this problem, we also used the national inpatient register for outcome assessment. We unfortunately also did not have access to the migration or death register. Analysis of responders and non-responders showed that those who had received in-patient psychiatric care were less likely to participate, thus leading to a selection bias limiting the generalizability of the study.[22] We do not know if the level of severity or treatment and confounders changed in the periods between the measurement points W1, W2 and W3. This might have resulted in over- or under-estimation of the associations. Although the cohort design we do not know the exact timing of the outcome events, and hence the incidence rate ratios could not be calculated. However, as the outcomes were not very common during follow-up we think the ORs have not been distorted and can be interpreted as risks. There might also have been some participants who developed depression in the period between W2 and W3. Although this study was able to adjust for common mediators and confounders, it did not offer the possibility to adjust for dyslipidemia, blood glucose, type and use of antidepressants and their compliance due to lack of such data.

## Conclusion

This study found that level of severity of depression seems to be significant for the risk of CVD, although not in a dose-response manner which might be obscured due to treatment. Overall, moderate depression showed the highest risk for CVD. Further, we found that concomitant anxious distress seems to imply a higher risk for CVD. Thus, concomitant anxious distress is not just a marker for an increased risk of a worse prognosis of depression but also a marker for a future risk of CVD, which stresses the need to always include anxious distress in assessments of depressed patients.

## Supporting Information

S1 Table Appendix A. DSM V criteria for anxious distress and corresponding questions used from scales in mental health in the PART study to assess anxious distress.
(DOC)
