# Supplementary material for: Severity of Depression, Anxious Distress and the Risk of Cardiovascular Disease in a Swedish Population-Based Cohort
Source: PLoS One. 2015 Oct 15;10(10):e0140742. doi: 10.1371/journal.pone.0140742 (PMC4607409; doi:10.1371/journal.pone.0140742)
Supplement: S1 Table — DSM V criteria for anxious distress and corresponding questions used from scales in mental health in the PART study to assess anxious distress. (DOC) [file pone.0140742.s001.doc]

Appendix A. DSM V criteria for anxious distress and corresponding questions used from scales in mental health in the PART study to assess anxious distress

| DSM V criteria for anxious distress ^1^ | Scale | Question on Symptom |
| --- | --- | --- |
| Feeling keyed up or tense. | Psychological well-being scale^2^ | How many times have you felt calm and relaxed during the past week?  Those who replied “never” or “sometimes” were regarded as having anxious distress symptom |
| Feeling unusually restless. | Major depression inventory ^3^ | How many times have you felt very restless in past 2 weeks?  Those who answered “all the time”, “most of the time” or “slightly more than half of the time” were regarded as having anxious distress symptom |
| Difficulty concentrating because of worry. | Major depression inventory | How many times have you had difficulty in concentrating in past 2 weeks?  Those who answered “all the time”, “most of the time” or “slightly more than half of the time” were regarded as having anxious distress symptom |
| Fear that something awful may happen. | Symptoms of anxiety during the past 30 days.^4^ | How much in the past 1 month have you experienced fear of dying?  Those who answered “much” or  “very much” were regarded as having anxious distress symptom |
| Feeling that the individual might lose control of himself or herself. | Symptoms of anxiety during the past 30 days | How much in the past 1 month have you had fear of losing control?  Those who answered “much” or “very much” were regarded as having anxious distress symptom |

^1^ Anxious distress is defined as the presence of at least two of the following symptoms during the majority of days of a major depressive episode or persistent depressive disorder (dysthymia). In the present study severity was classified as; mild: two symptoms, moderate: three symptoms, moderate-severe: four or five symptoms; and severe: four or five symptoms and with motor agitation. (http://dsm.psychiatryonline.org//, doi: 10th April 2015)

^2^ Bech, P., Gudex, C., Staehr Johansen, K. (1996). The WHO (Ten) Well-Being Index: Validation in Diabetes. Psychother Psychosom, 65, 183-190.

^3^ Bech, P., Wermuth, L. (1998). Applicability and validity of the Major Depression Inventory in patients with Parkinson´s disease. Nord J Psychiatry, 52, 305-309

.^4^ Sheehan, D. V. (1983). The anxiety disease (pp 124-129). New York: Charles Scribners Sons
